# Characterization of Portuguese Centenarian Eating Habits, Nutritional Biomarkers, and Cardiovascular Risk: A Case Control Study

**DOI:** 10.1155/2018/5296168

**Published:** 2018-03-13

**Authors:** A. Pereira da Silva, A. Valente, C. Chaves, A. Matos, A. Gil, A. C. Santos, J. P. Gorjão-Clara, M. Bicho

**Affiliations:** ^1^Alameda Primary Care Health Center, Ministry of Health, Lisbon, Portugal; ^2^Genetics Laboratory Environmental Health Institute (ISAMB), Faculty of Medicine, University of Lisbon, Lisbon, Portugal; ^3^Geriatric Universitary Unit of Faculty of Medicine, University of Lisbon, Lisbon, Portugal; ^4^Atlântica-School of Management Sciences, Health, IT & Engineering, Fábrica da Pólvora de Barcarena, 2730-036 Barcarena, Portugal; ^5^Centro Hospitalar de Lisboa Central, Lisboa, Portugal; ^6^Instituto de Investigação Científica Bento da Rocha Cabral, Lisbon, Portugal; ^7^Academic Medical Center of Lisbon-North of Lisbon Hospital Center, Lisbon, Portugal

## Abstract

**Background and Aims:**

Eating habits may contribute to longevity. We characterized the eating habits and cardiovascular risk (CVR) biomarkers in Portuguese centenarians (CENT) compared to controls.

**Methods and Results:**

Centenarians (*n* = 253), 100.26 ± 1.98 years, were compared with 268 controls (67.51 ± 3.25), low (LCR) and high (HCR) CVR (QRISK®2-2016). Anthropometric and body composition were evaluated by bioimpedance. Abdominal obesity, BMI, and fat mass (FM) cut-offs were according to the WHO. Sarcopenia was defined by muscle mass index cut-off ≤ 16.7 kg/m^2^. Daily red meat intake, adjusted for age and gender, was sarcopenia protective (OR = 0.25, 95% CI = 0.096–0.670, *P* = 0.006); however, it contributes for FM excess (OR = 4.946, 95% CI = 1.471–16.626, *P* = 0.01), overweight, and obesity (OR = 4.804, 95% CI = 1.666–13.851, *P* = 0.004). This centenarian eating habit (2%) contrasts to HCR (64.3%). The history of red meat (*P* < 0.0001) and canned/industrialized food intakes (*P* < 0.0001) was associated with HCR. Basal metabolism was lower in centenarians versus LCR/HCR (CENT = 1176.78 ± 201.98; LCR = 1356.54 ± 170.65; HCR = 1561.33 ± 267.85; *P* < 0.0001), BMI (CENT = 21.06 ± 3.68; LCR = 28.49 ± 4.69; HCR = 29.56 ± 5.26; *P* < 0.0001), waist circumference (CENT = 85.29 ± 10.83; LCR = 96.02 ± 11.71; HCR = 104.50 ± 11.84; *P* < 0.0001), and waist-hip ratio (CENT = 0.88 ± 0.07; LCR = 0.92 ± 0.08; HCR = 1.01 ± 0.08; *P* < 0.0001). CENT had lower total cholesterol, LDL cholesterol, non-HDL cholesterol, and cholesterol/HDL ratio than controls.

**Conclusions:**

Frequent consumption of red meat, cholesterol, and heme iron rich may contribute to obesity and increased CVR. The low frequency of this consumption, observed in centenarians, although associated with sarcopenia, may be one of the keys to longevity.

## 1. Introduction

According to the WHO, very old individuals are a rapidly growing age group around the globe, thanks to the improvements in medicines, as well as the modification lifestyle. This nutrition characteristic is a key component for achieving good health [1]. Adults approaching 70 years will more likely face problems of caloric excess, leading to overweight or obesity [2].

There are several methods to evaluate the eating habits [3]. Retrospective methods are a good tool for assessing past eating habits [4]; however, they have some limitations, particularly in populations such as the elderly and children groups [3]. Photographic models may play an important role when used in conjunction with retrospective methods of food intake assessment [5]. In epidemiological studies, the choice of method to use depends on many factors. The food frequency questionnaire (FFQ) is a method regularly used in epidemiological studies. Its use makes possible to evaluate the habitual frequency of food consumption over longer periods of time. It is considered the most practical and informative method to evaluate the relation of causality between food consumption and disease [6]. The structure of the FFQ is usually composed of a predefined food list and a section with the frequency of consumption. Some FFQs are semiquantitative, defining a mean reference portion consumed, so that individual reports define whether their consumption was higher, equal, or lower than the average portions presented in home measures [7].

Findings from a meta-analysis indicate that high consumption of red meat, in particular, processed meat, is associated with higher all-cause mortality [8]. Epidemiologic studies have linked the consumption of red or processed meat with obesity, type 2 diabetes, cardiovascular disease (CVD), and cancer [9, 10]. A meta-analysis of 12 cohort studies showed a 20% increase risk of diabetes per 120 g/day increase in red meat intake and, for processed red meat, a 57% increase risk per 50 g/day increase [11].

Adipose tissue is an active endocrine organ that effects insulin sensitivity and production of insulin-like growth factors and increases the oxidative stress and chronic low-grade inflammation affecting immune response [12]. In obesity, increased release from the adipose tissue of free fatty acids, TNF-*α*, and resistin and reduced release of adiponectin lead to the development of insulin resistance. Cancer death rates increase, mostly as a consequence of the aging of the population. A healthy diet and control of obesity based on abundant and variable plant foods, high consumption of cereals, olive oil as the main fat, low intake of red meat, and moderate consumption of wine reduced the risk of CVD and cancer [13].

The pathophysiology of sarcopenia is complex, having not modifiable contributory factors, including the aging process, leading to reduced sex hormones and mitochondrial dysfunction [14]. In addition, some subjects will experience neurodegenerative disease with aging that will have detrimental effects in terms of muscle signaling and function [15]. Increases in fat mass may contribute to the loss of muscle mass that ultimately leads to sarcopenic obesity through increased inflammation and upregulation of protein degradation via the ubiquitin-proteasome pathway [16].

In obesity, the presence of inflammatory factors may have detrimental effects on amino acid utilization and/or insulin signaling pathways involved in the stimulation of muscle synthesis following food intake [17].

The physiological and morphological changes in skeletal muscle with advancing age are characterized by overall declines in size and number of skeletal muscle fibers, mainly the type 2 or fast-twitch muscle fibers, and a marked infiltration of fibrous and adipose tissue into the skeletal muscle [18].

There is a physiological decline in food intake with aging. The reasons are multifactorial (interindividual variations) and may include alterations in the hedonic qualities of food (decreased odor and taste sensations), increased gastrointestinal satiation signals, and a decline in the central feeding drive [19]. The type of diet and eating habits may determine, throughout a nutrigenetic interaction, the levels of reactive species, oxidative stress, and chronic disease development, namely, cardiovascular ones [20]. Nutrients affecting gene expression and genomic integrity modulate disease processes such as cancer, cardiovascular disease, and neurodegenerative disorders [21]. The high consumption of red meat, saturated fatty acids, and cholesterol may be associated with increased risk of diabetes, CVD, and mortality risk [22]. Free radicals and neuroinflammation processes underlie many neurodegenerative conditions [23]. The diets identified as Alzheimer's disease protectors were associated with higher intake of vegetables, fruit, whole grains, fish, and legumes and with lower intake of high-fat dairies, processed meat, and sweets [24]. Currently, besides nutrition longevity influence via complex epigenetic mechanisms [25], emerging research techniques such as nutrigenomics, metabolomics, and proteomics indicate that the type of food and dietary restriction can lead to cell health status capable of modulating apoptosis, reactive oxygen species and reactive nitrogen species detoxification, and gene response, towards disease prevention and longevity [23].

For all these reasons and because there are still no studies in all Portuguese population on this field, we went to characterize the eating habits and nutritional and cardiovascular biomarkers from Portuguese centenarians, to compare them with both high and low cardiovascular risk (CVR) controls.

## 2. Methods

### 2.1. Study Patients

We studied from 2012 to 2015 a total of 521 subjects, both genders, being 253 centenarians (CENT) (100.26 ± 1.98 years old) 197 women (77.9%) and 56 men (22.1%). The control group included 268 subjects (67.51 ± 3.25 years old), being 164 women (61.2%) and 104 men (38.8%). This group had both low (LCR) and high cardiovascular risks (HCR); calculations were based on QRISK2-2016 [26]. Centenarians, from all the regions of Portugal, were identified, enrolled, and evaluated at their usual place of residence, as previously described [27]. Centenarian individuals, although uniformly distributed throughout the country, predominated in the Castelo Branco District, followed by Lisbon. The area of Castelo Branco District, surrounded by mountains in the orographic aspect, is mainly rural. On the other hand, the area of Lisbon is mainly an urbanized area. At the time of the interview, most of the centenarians (69.2%) reported having lived most of the life in the interior of the country and only 30.8% in coastal regions. Most of them (51%) lived in small villages for most of their life, but it is noteworthy that one part (30.4%) reported having lived in a city environment. Although all the centenarian individuals presented a capacity for understanding and communication (being an exclusion criterion otherwise), the centenarian men of the present study presented cognitive scores superior to those of centenarian women. The control group included patients recruited from the Heart and Vessels Department of Santa Maria Hospital and from a primary health care center in Lisbon, Portugal. Hospital de Santa Maria is a reference hospital at the national level, and as such, the controls are not all of the Lisbon region but of several regions of the country.

### 2.2. Nutrition Data

Anthropometric and body composition analyses were evaluated by bioimpedance, using a portable tetrapolar bioelectrical equipment, the Tanita® BC-420MA (Tanita Corporation of America Inc., Illinois, USA) device to estimate weight, body mass index (BMI), fat mass (FM), muscle mass (MM), and resting metabolic rate (RMR). The MM and FM indexes were calculated [kg/height (m^2^)]. Exclusion criteria for bioimpedance measurements were previously described [27].

Data were collected by applying a semiquantitative food frequency questionnaire, based on a validated FFQ for a Portuguese population [28]. The questionnaire used was composed of a list of food groups with 10 items (red meat, fish, eggs, sweets, dairy products, vegetables, legumes, fruits, oilseeds, and canned food) and one closed section with five categories of frequencies of consumption. A photographic manual was used, published by the Institute of Public Health Dr. Ricardo Jorge, I.P., [29] as a visual support for the identification of multiples and submultiples of the middle portion. Data were statistically analyzed in order to know the differences of consumption of food groups between the centenarians and the control group of both high and low CVR.

### 2.3. Biomarkers and Cardiovascular Risk

Participants or their direct supervisors were asked to provide access to the latest routine blood analyses. The following biochemical data, obtained by laboratory routine analysis measured in certified labs, were collected when available: glucose, total cholesterol (TC), LDL-C, HDL-C, non-HDL-C, triglycerides (TG), uric acid, urea, and creatinine, or calculated: non-HDL-C.

Dyslipidemia was defined when one of the following conditions was present: TC ≥ 200 mg/dL, TG ≥ 150 mg/dL, LDL-C ≥ 100 mg/dL, and HDL-C ≤ 40 mg/dL in men or ≤50 mg/dL in women [30].

The abdominal obesity (cm), BMI (kg/m^2^), and the cut-off for FM by gender were established in agreement with WHO guidelines [31]. Sarcopenia was defined by muscle mass index cut-off ≤ 16.7 kg/m^2^ [32].

CVR was calculated using a QRISK 2-2016 risk calculator program (https://qrisk.org), based on age, gender, ethnicity, smoking habits, diabetes status, angina or heart attack in a 1st degree relative age below 60 years, chronic kidney disease (stage 4 or 5), atrial fibrillation, hypertension, and rheumatoid arthritis and also based on cholesterol/HDL ratio, systolic blood pressure, and body mass index [26, 33].

### 2.4. Ethical Considerations

This study was approved by the Scientific and Ethics Committees of the Lisbon Academic Medical Centre (Faculty of Medicine of the University of Lisbon and Santa Maria Hospital) and by the National Commission for Data Protection and was conducted in agreement with the Helsinki Declaration. All the participants gave their written informed consent in order to be included in the study.

### 2.5. Statistical Analysis

Statistical analysis was performed using the computer software for Windows SPSS, version 20.0 (SPSS Inc., Chicago). The results of quantitative variables were expressed as the mean ± standard deviation and for qualitative categorical variables as the number and percentage. To test the normality of all variables, the Kolmogorov-Smirnov test was applied. Categorical variables were compared with the chi-square with the Z-proportion test or Mann–Whitney *U* test. The comparison of means between groups of numeric variables and normally distributed means was performed by one-way analysis of variance (ANOVA) or Kruskal-Wallis test, followed by the Tukey test. The values of nonnormal parameters are presented in median and interquartile range. Numeric variables were related by the application of Pearson or Spearman correlation coefficients. Binary and multivariate logistic regression analysis was performed. As the measure of association, the odds ratio (OR) was used with the respective 95% confidence interval. All the tests were considered statistically significant if *P* < 0.05.

## 3. Results

**Figure 1:**
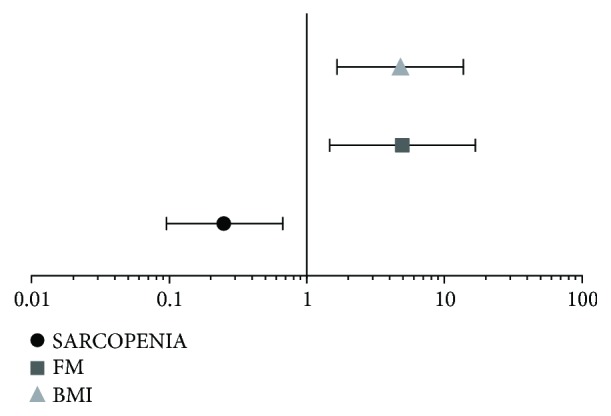
Odds ratio of daily intake of red meat, adjusted for age and gender concerning sarcopenia, fat mass excess, and overweight/obesity. The *x*-axis is in logarithmic scale (log 10).

**Figure 2:**
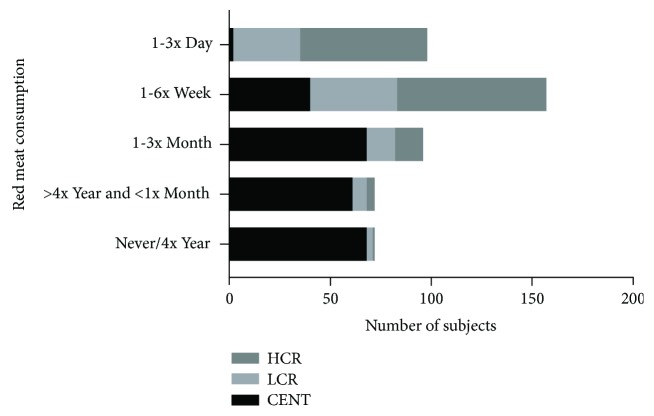
Distribution of the frequencies of red meat intake during most of the life among the groups: centenarians (CENT), low cardiovascular risk (LCR) control group, and high cardiovascular risk (HCR) control group.

There were differences in the frequency of food groups' consumption between centenarians and controls, except for the oilseed group (see Table 1). As shown in Figure 1, the daily intake of red meat, adjusted for age and gender, was a protective factor for sarcopenia (OR = 0.25, 95% CI: 0.096–0.670, *P* = 0.006); however, it contributes for FM excess (OR = 4.946, 95% CI: 1.471–16.626, *P* = 0.01), overweight, and obesity (OR = 4.804, 95% CI:1.666–13.851, *P* = 0.004). Only 2% of the centenarians reported this eating habit in the opposite 64.3% of the HCR group. In Figure 2, we can see that the frequency history of red meat intake was associated with higher CVR (*χ*^2^ = 239.807; df = 8, *P* < 0.0001), in the same way of canned food intake (*χ*^2^ = 225.321; df = 8, *P* < 0.0001).

**Figure 3:**
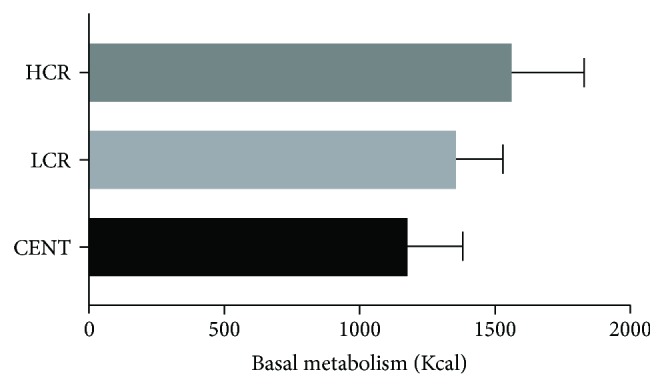
Basal metabolism of all groups: centenarians (CENT), low cardiovascular risk (LCR) control group, and high cardiovascular risk (HCR) control group.

**Figure 4:**
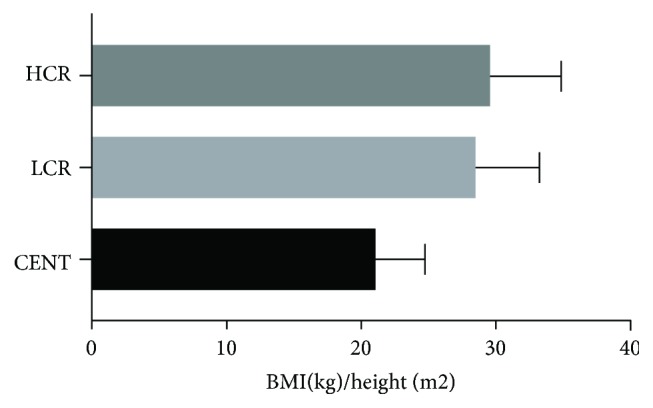
BMI (kg)/height (m^2^) of all groups: centenarians (CENT), low cardiovascular risk (LCR) control group, and high cardiovascular risk (HCR) control group.

**Figure 5:**
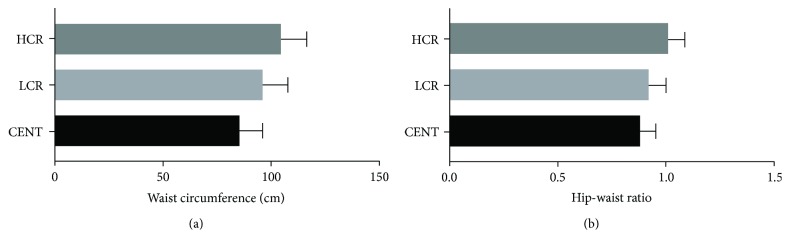
(a) Waist circumference (cm) of all groups: centenarians (CENT), low cardiovascular risk (LCR) control group, and high cardiovascular risk (HCR) control group. (b) Waist-hip ratio of all groups: centenarians (CENT), low cardiovascular risk (LCR) control group, and high cardiovascular risk (HCR) control group.

Basal metabolism (Kcal) was lower in centenarians and higher in the HCR group (Figure 3) (CENT = 1176.78 ± 201.98 versus LCR = 1356.54 ± 170.65 versus HCR = 1561.33 ± 267.85; *P* < 0.0001). Compared with controls, centenarians also had a lower BMI (CENT = 21.06 ± 3.68 versus LCR = 28.49 ± 4.69 versus HCR = 29.56 ± 5.26; *P* < 0.0001) (Figure 4), waist circumference (cm) (CENT = 85.29 ± 10.83 versus LCR = 96.02 ± 11.71 versus HCR = 104.50 ± 11.84; *P* < 0.0001) (Figure 5(a)), and waist-hip ratio (CENT = 0.88 ± 0.07 versus LCR = 0.92 ± 0.08 versus HCR = 1.01 ± 0.08; *P* < 0.0001) (Figure 5(b)).

Considering the biochemical parameter values of CVR, particularly, lipidogram and lipid profile, there were significant differences between the results obtained between the group of centenarian individuals and those of the low-risk and high-risk control groups (Table 2).

Total cholesterol (*P* < 0.0001), LDL-C (*P* < 0.0001), and non-HDL cholesterol (*P* < 0.0001) levels were lower in the centenarian group and differed significantly from either the low or the high cardiovascular risk control subgroups (Table 2).

In relation to LDL values, there were no significant differences between LCR and HCR subjects (*P* = 0.161, Tukey test). In cholesterol/HDL cholesterol ratio, there was no significant difference between centenarians and LCR subjects (*P* = 0.960, Tukey test) (Table 2).

## 4. Discussion

As far as we know, this is an original work in human longevity which investigates some aspects of eating habits, anthropometry, basal metabolism, and blood parameters. We sought to know the history of the eating habits of Portuguese centenarians and verify if these habits were or not coincident with the history of the dietary profile of younger individuals, some of them with HCR and others with LCR, whose probable life expectancy, according to the projection of the 2011 census, does not exceed 84 years [34].

We applied a semiquantitative food frequency questionnaire using photographic models because it was considered to be the most appropriate for the population studies [35, 36]. The 24-hour questionnaire is a retrospective method considered the one with the best accuracy to estimate food intake [37]. However, as mentioned in epidemiological studies in the elderly, the required repetition of the previous 24-hour questionnaire may be more inaccurate in comparison with a food frequency questionnaire in which participants report their past eating habits in a single interview. A large part (71.9%) of the centenarians studied was institutionalized so the present eating habits were very different from the past ones. In the centenarians, it was possible to observe the difference in the ease to recall past eating habits in relation to the most recent ones.

The food history showed that the frequency of consumption of legumes, fruits, and vegetables is higher and red meat consumption is lower in the centenarians compared to the control group. The latter was frequently ingested with larger and repeated food portions (Table 1). Both aspects are indicative that the daily caloric intake of the centenarians may be lower than that of the controls and that by consuming foods with health benefits (vitamins, bioactive compounds, and dietary fiber) more often supports the idea that can promote longevity (Figure 2). These data may lead to a reflection on the importance of eating habits such as caloric overload and in particular that associated with red meat ingestion in longevity.

The centenarians had distinguished themselves from controls in all food groups that have been evaluated with the exception of oilseed ingestion. There are studies that indicate the excess consumption of red meat as a negative impact related to good health since this consumption was associated with obesity, type 2 diabetes, CVD, cancer [10], and higher all-cause mortality [8], and accumulating scientific evidence has indicated that high consumption of red meat, especially processed meat, may be associated with an increased risk of major chronic diseases [22].

We found that the individuals with the highest CVR were those who had the highest frequencies of red meat consumptions (Figures 2, 5(a), and 5(b)). In fact, this consumption, in particular, processed meat, is associated with a higher incidence of CVD such as coronary heart disease, heart failure, and stroke in addition to other pathologies [22].

Red meat, on the other hand, is a source of heme iron [38]. Free heme may catalyze oxidant processes involving several components of biological systems, resulting in tissue damage and ultimately leading to disease. Actually, heme-catalyzed oxidations can damage lipids, proteins, DNA, and other nucleic acids and various components of biological systems. A major pathway involves reactions of lipids with heme: LOOH (lipid hydroperoxide) + Fe-ligands (heme) ➔ LOOFe ligands ➔ LO^∗^ (lipid alkoxy radical) + ^∗^OFe ligands (heme oxyradical). The alkoxy radicals and the heme oxy radicals can initiate further oxidations some of which would result in oxidative chain reactions. Heme catalysis of oxidation is the strongest oxidizing system for developing tissue damage. These heme-catalyzed oxidations can lead to the initiation of biochemical and cellular damage and subsequently disease processes [39]. Also, the formation of *N-nitroso* compounds in the intestine conditioned by the ingestion of red meat may lead to oxidative stress and DNA damage [40]. High red meat consumption was associated with modestly higher concentrations of plasma GGT and hs-CRP, whereas high whole grain bread consumption was related to modestly lower concentrations of GGT, ALT, and hs-CRP [41]. The association of red meat consumption with increased levels of hs-CRP could be modified by high whole grain bread consumption [41].

These facts highlighted the hypothesis that dietary factors may modulate these biomarkers, which may be potential mediators related to the risk of diabetes and CVD [41]. Even more, the discovery of a link between L-carnitine ingestion, gut microbiota metabolism, and CVD risk revealed a new pathway linking dietary red meat ingestion with atherosclerosis pathogenesis pointing out the role of gut microbiota in this pathway suggesting a new potential therapeutic target for preventing CVD [42].

Red meat is known to have higher contents of saturated fat and cholesterol [38]; this fact agrees with our observations revealing that centenarians (24.1%) have low hypercholesterolemia frequency than controls of low (75.8%) and high (78.9%) CVR. Additionally, the cholesterol/HDL ratio was statistically higher (*P* = 0.017) in the high-risk subgroup (4.24 ± 1.18) compared to centenarians (3.81 ± 1.09) (Table 2). We assumed that the centenarians have low CVR since they reached extreme longevity. We observed that they differ from the other groups, namely, the HCR group having lower values of total cholesterol, LDL cholesterol, non-HDL-C, and cholesterol/HDL ratio. LDL-C and non-HDL-C are atherogenic factors, the latter including TG-rich lipoproteins, cholesteryl ester-enriched remnants of TG-rich lipoproteins, and lipoprotein(a) with great predictive CVR value [43].

Excess meat consumption was associated with an increase in fat mass, obesity, and waist circumference and increased waist-hip ratio associated with the HCR group (Figures 4, 5(a), and 5(b)). As observed for red meat intake, our results support this observation, since the frequency (at least 1x a week) of consumption of red meat (*χ*^2^ = 239.807; df = 8, *P* < 0.0001) as well as canned/industrialized foods (*χ*^2^ = 225.321; df = 8, *P* < 0.0001) was associated with HCR individuals compared to the other groups.

Similarly to that observed with red meat, a higher frequency of canned/industrialized food consumption in HCR individuals compared to LCR and centenarians (72.3% versus 25.5% versus 2.1%, resp., consumed at least 1x per week) was observed. It is known that polyphosphates are commonly used as an additive in industrially processed food and may increase serum phosphate levels leading to vascular damage and cardiovascular morbidity inducing aging processes [44].

Concerning meat consumption, however, we found a benefit in relation to a possible contributor to prevent sarcopenia, as verified by Rondanelli et al. [45]. The underlying cause of sarcopenia is unclear but may include a lower basal rate of protein synthesis in aged muscle. Meats are nutrient-rich sources of protein which are potently stimulatory for muscle protein synthesis and may aid in mediating gains in muscle mass and strength when combined with exercise program [46].

Although beneficial for the prevention of sarcopenia, however, red meat consumption may increase the risk of stroke. In fact, red meat is a source of saturated fatty acids and cholesterol. Some studies have indicated that a high intake of saturated fatty acids increases total cholesterol levels, LDL, and triglycerides, which could increase the risk of stroke [47]. No sarcopenic obesity was observed either in the controls or in the centenarians, which were mostly eutrophic.

It was verified that the group of centenarians consumed more vegetables/legumes/fruits than the control groups (HCR and LCR, Table 1) that may contribute to longevity. Epidemiological studies suggest a role of fruits and vegetables, in protection against disease of aging [23], and the WHO considers that these should be the main foods to be ingested [48]. Actually, the exogenous antioxidants, greatly relevant for longevity, such as vitamin C (ascorbic acid/ascorbate), vitamin E (tocopherols, tocotrienols), carotenoids (*α*-carotene, *β*-carotene, zeaxanthin, lutein, lycopene, *β*-cryptoxanthin, etc.), polyphenols (flavonols, flavanols, anthocyanins, isoflavones, and phenolic acid), and trace elements (selenium, zinc), predominate in dietary sources derived mainly from the vegetable kingdom [20].

It must be considered protein supplementation in patients with sarcopenia with no medical contraindications [49], which can contribute to improve not only the muscular mass but also the cognitive aspects [50].

The basal metabolism decreases with age [51], which was also observed in our study. It was found that individual HCR controls had an increased basal metabolism compared to the other groups (LCR and centenarians). We assume that the centenarians had a low CVR profile; otherwise, they would not have reached that age.

## 5. Study Strengths and Limitations

In this case-control study design, the sample size is adequate according to what has been previously explained [27]. The group of centenarians is compared with a group of younger individuals assuming that the probability of reaching 100 years is remote for the control group. On the other hand, it is also assumed that the CVR of centenarians is small compared to the control group since otherwise they would not have reached 100 years. Estimation of energy and nutrient intake may be considered a study limitation although the frequency of consumption and food portion size were evaluated.

## 6. Conclusions

Centenarians have a different food history than the control population. Frequent consumption of red meat contributes to obesity and increased CVR, since LDL-cholesterol and heme iron of red meat that catalyze oxidations may lead to atherosclerosis disease processes. Menus mainly with vegetables and legumes and less red meat, observed in centenarians, although associated with sarcopenia, may promote a longer life span.

## Figures and Tables

**Table 1:** Frequency of food consumption and comparison between centenarians (CENT) and low (LCR) and high (HCR) cardiovascular risk control groups. The amount/day and repetition refers to the main meal.

		LCR, *n* (%)	HCR, *n* (%)	CENT, *n* (%)	*P* value
Number of meals/day	1–3	52 (15.5)	99 (29.5)	185 (55.1)	<0.0001
	4-5	46 (34.3)	43 (32.1)	45 (33.6)	
	6 or more	1 (16.7)	5 (83.3)	0 (0.0)	

Amount/day	Mini	6 (4.1)	5 (3.4)	136 (92.5)	<0.0001
	Medium	70 (30)	89 (38.2)	74 (31.8)	
	Full	19 (26.4)	38 (52.8)	15 (20.8)	
	Very full	3 (21.4)	10 (71.4)	1 (7.1)	

Repetition	No	73 (20.3)	94 (26.2)	192 (53.5)	<0.0001
	Yes	24 (25.3)	46 (48.4)	25 (26.3)	

Red meat	Never/4x a year	3 (4.2)	1 (1.4)	68 (94.4)	<0.0001
	>4x a year, <1x a month	7 (9.7)	4 (5.6)	61 (84.7)	
	1–3x a month	14 (14.6)	14 (14.6)	68 (70.8)	
	1–6x a week	43 (27.4)	74 (47.1)	40 (25.5)	
	1–3x a day	33 (33.7)	63 (64.3)	2 (2)	

Fish	Never/4x a year	1 (2.6)	1 (2.6)	36 (94.7)	<0.0001
	>4x a year, <1x a month	2 (2.7)	1 (1.3)	72 (96)	
	1–3x a month	12 (12.4)	25 (25.8)	60 (61.9)	
	1–6x a week	68 (28.5)	108 (45.2)	63 (26.4)	
	1–3x a day	17 (37.8)	21 (46.7)	7 (15.6)	

Eggs	Never/4x a year	4 (16.7)	5 (20.8)	15 (62.5)	0.009
	>4x a year, <1x a month	17 (18.3)	23 (24.7)	53 (57)	
	1–3x a month	42 (26.6)	60 (38)	56 (35.4)	
	1–6x a week	35 (17.7)	62 (31.3)	101 (51)	
	1–3x a day	2 (20)	6 (60)	2 (20)	

Sweets	Never/4x a year	11 (8.1)	21 (15.6)	103 (76.3)	<0.0001
	>4x a year, <1x a month	11 (13.6)	24 (29.6)	46 (56.8)	
	1–3x a month	33 (33.7)	29 (29.6)	36 (36.7)	
	1–6x a week	27 (23.5)	51 (44.3)	37 (32.2)	
	1–3x a day	18 (28.1)	30 (46.9)	16 (25)	

Dairy	Never/4x a year	4 (26.7)	3 (20)	8 (53.3)	0.001
	>4x a year, <1x a month	2 (4.7)	10 (23.3)	31 (72.1)	
	1–3x a month	8 (18.2)	10 (22.7)	26 (59.1)	
	1–6x a week	19 (22.6)	18 (21.4)	47 (56)	
	1–3x a day	67 (21.9)	114 (37.3)	125 (40.8)	

Vegetables	Never/4x a year	1 (50)	1 (50)	0 (0)	<0.0001
	>4x a year, <1x a month	1 (50)	1 (50)	0 (0)	
	1–3x a month	4 (23.5)	11 (64.7)	2 (11.8)	
	1–6x a week	35 (36.1)	58 (59.8)	4 (4.1)	
	1–3x a day	59 (15.6)	84 (22.3)	234 (62.1)	

Legumes	Never/4x a year	1 (20)	3 (60)	1 (20)	<0.0001
	>4x a year, <1x a month	13 (50)	12 (46.2)	1 (3.8)	
	1–3x a month	40 (38.1)	60 (57.1)	5 (4.8)	
	1–6x a week	40 (26.3)	70 (46.1)	42 (27.6)	
	1–3x a day	6 (3.1)	10 (5.1)	180 (91.8)	

Fruits	Never/4x a year	2 (100)	0 (0)	0 (0)	0.040
	>4x a year, <1x a month	0 (0)	4 (66.7)	2 (33.3)	
	1–3x a month	3 (13.6)	10 (45.5)	9 (40.9)	
	1–6x a week	16 (15.4)	34 (32.7)	54 (51.9)	
	1–3x a day	79 (22.1)	107 (29.9)	172 (48)	

Oilseeds	Never/4x a year	30 (17.3)	53 (30.6)	90 (52)	0.401
	>4x a year, <1x a month	28 (21.9)	38 (29.7)	62 (48.4)	
	1–3x a month	23 (22.5)	37 (36.3)	42 (41.2)	
	1–6x a week	15 (25.9)	23 (39.7)	20 (34.5)	
	1–3x a day	4 (22.2)	4 (22.2)	10 (55.6)	

Canned	Never/4x a year	25 (9)	41 (14.7)	212 (76.3)	<0.0001
	>4x a year, <1x a month	29 (37.2)	32 (41)	17 (21.8)	
	1–3x a month	31 (40.8)	40 (52.6)	5 (6.6)	
	1–6x a week	12 (25.5)	34 (72.3)	1 (2.1)	
	1–3x a day	3 (25)	9 (75)	0 (0)	

**Table 2:** Lipid profile comparison between centenarians (CENT) and low (LCR) and high (HCR) cardiovascular risk control groups.

	CENT	LCR	HCR	*P*
Total cholesterol (mg/dL)	178.81 ± 42.36^a,b^	213.51 ± 46.98	194.84 ± 42.91^a^	<0.0001
HDL cholesterol (mg/dL)	47.00 [38.00–56.00]^a^	56.00 [49.00–67.00]^b^	47.00 [38.00–55.00]	<0.0001^∗^
Triglycerides (mg/dL)	106.00 [86.00–134.30]^a^	94.00 [71.00–133.00]^b^	117.00 [91.00–156.50]	0.001^∗^
LDL cholesterol (mg/dL)	96.4 [78.5–129.0]^a,b^	123.8 [104.7–151.17]	122.3 [93.6–145.85]	<0.0001^∗^
Non-HDL cholesterol (mg/dL)	127.33 ± 38.75^a,b^	153.08 ± 43.29	145.99 ± 39.95	<0.0001
Ratio total cholesterol/HDL cholesterol	3.71 [3.02–4.41]^b^	3.66 [3.06–4.16]^b^	4.03 [3.36–4.89]	0.001^∗^

^a^Different from LCR. ^b^Different from HCR. ^∗^Results expressed in median [IQR 25–75]. Kruskal Wallis Test was used.
